# Aortic Valve Replacement for Asymptomatic Severe Aortic Stenosis: A New Management Paradigm?

**DOI:** 10.1016/j.jscai.2025.103721

**Published:** 2025-05-07

**Authors:** Aravdeep Jhand, Islam Y. Elgendy

**Affiliations:** a Department of Cardiology, Geisinger Medical Center, Danville, Pennsylvania; b Division of Cardiovascular Medicine, Gill Heart and Vascular Institute, University of Kentucky, Lexington, Kentucky

**Keywords:** aortic stenosis, early intervention, surveillance

Aortic stenosis (AS) is one of the most prevalent valvular heart diseases in developed countries. With life expectancy increasing, the global burden associated with severe AS is expected to rise.^1^ The natural history of AS involves gradual yet unpredictable calcific degeneration of the aortic valve, leading to progressive hemodynamic obstruction and subsequent risk of irreversible left ventricular (LV) remodeling, heart failure, and sudden cardiac death.^2^ Symptom development represents an important watershed period in the clinical course of patients with AS. The risk of death in asymptomatic severe AS is estimated to be 1% to 2% per year, which may dramatically increase up to 4% in the first 3 months after symptom onset.^3^ While symptomatic severe AS is a class I indication for aortic valve replacement (AVR), the American and European guidelines recommend AVR for asymptomatic patients only under certain circumstances, which include LV dysfunction (ejection fraction <50%), undergoing concomitant cardiac surgery, abnormal exercise stress test, or increase in aortic valve velocity of >0.3 m/s over a year.^4^^,^^5^

Clinical surveillance (CS) is believed to be a safe strategy for the majority of patients with asymptomatic severe AS because the risk of intervention may outweigh the potential risk of adverse events. Several concerns have been raised by the proponents of earlier intervention.^6^ These concerns include loss of follow-up, delay in access to intervention once symptoms develop, lack of any meaningful medical therapy that regresses AS progression, and development of irreversible LV dysfunction. Moreover, assessment of symptoms in certain patients with mobility limitations or other comorbidities may be challenging, and stress testing might not be feasible for all patients.

Interest in evaluating the role of early AVR in asymptomatic severe AS has been met with both enthusiasm and skepticism alike, especially with widespread adoption of less invasive transcatheter aortic valve replacement (TAVR). This has culminated in 4 randomized controlled trials (RCTs). Two small trials (RECOVERY and AVATAR) demonstrated that a strategy of early surgical aortic valve replacement (SAVR) reduced the risk of operative mortality or death from cardiovascular cause and the composite of all-cause death, acute myocardial infarction, stroke, or unplanned hospitalization for heart failure, respectively.^7^^,^^8^ One trial (EVOLVED) enrolled patients who underwent a mixture of SAVR (75%) and TAVR (25%) in the early intervention arm and showed no difference in terms of the primary composite outcome of all-cause death or unplanned AS-related hospitalization.^9^ The largest trial (EARLY TAVR) showed that TAVR reduced the composite outcome of death, stroke, or unplanned hospitalization, driven by a reduction in hospitalizations for cardiovascular causes.^10^ In 3 trials, asymptomatic status was confirmed with low-level stress tests,^7^^,^^8^^,^^10^ whereas in the EVOLVED trial, it was based on LV hypertrophy on electrocardiogram, elevated troponin levels, and evidence of myocardial fibrosis on cardiac magnetic resonance imaging.^9^ Notably, conversion to AVR in the CS group was variable, ranging from a median of 11.1 months in EARLY TAVR to 700 days in RECOVERY, and a significant number of patients enrolled in the CS arm received AVR during follow-up (ranging from 31.6% in AVATAR to 87% in EARLY TAVR). The delay to AVR was shortest in EARLY TAVR (median 32 days), highlighting the differences in the health care systems across the countries in which these RCTs were conducted (Table 1).

**Table 1:** Key characteristics and primary findings from randomized controlled trials comparing early aortic valve replacement vs clinical surveillance in patients with asymptomatic severe AS.

Trial	Countries involved	N	Mean age, y	AVR	CS	Definition of severe AS	Exercise testing performed	Type of intervention in AVR group	CS patients that required AVR	Time to intervention, median (IQR)		Follow-up duration, median (IQR)	Primary outcome	Key findings
										AVR	CS			
RECOVERY^7^	Korea	145	64.2	73	72	AVA < 0.75 cm^2^, AV PV > 4.5 m/s, or AV MG >50 mm Hg	8.8%	SAVR 100%	74%	23 d (10-36)	700 d (277-1469)	6.2 years (5-7.4)	Composite of operative mortality or death from cardiovascular causes	Primary outcome: 1% in AVR vs 15% in CS (HR, 0.09; 95% CI, 0.01-0.67)
AVATAR^8^	Belgium, Czech Republic, Italy, Croatia, Lithuania, Poland, and Serbia	157	67	78	79	AVA < 1 cm^2^, AV PV >4 m/s, or AV MG >40 mm Hg	100%	SAVR 100%	31.6%	55 d (36-79)	400 d (191-619)	32 months	Composite of all-cause mortality, acute myocardial infarction, stroke, and unplanned heart failure hospitalization	Primary outcome: 15.2% in AVR vs 34.7% in CS (HR, 0.46; 95% CI, 0.23-0.90; *P* = .02)
EVOLVED^9^	United Kingdom, Australia	226	73	113	111	AV PV >4 m/s or AV PV >3.5 m/s with AVAi of <0.6 cm^2^/m^2^	N/A	SAVR 75% TAVR 25%	77%	5 mo (3.4-8)	20.2 mo (11.4-42.0)	42 months	Composite of all-cause mortality or unplanned AS-related hospitalization	Primary outcome: 18% in AVR vs 23% in CS; (HR, 0.89; 95% CI, 0.44-1.43; *P* = .44)
EARLY TAVR^10^	United States, Canada	901	75.8	455	446	AVA < 1 cm^2^ or AVAi < 0.6 cm^2^/m^2^ and AV MG of 40 mm Hg or AV PV 4 m/s	90.6%	TAVR 100%	87%	14 d (9-14)	11.1 mo (5.0-19.7)	3.8 years (2.8-5.0)	Composite of death from any cause, stroke, or unplanned hospitalization for cardiovascular causes	Primary outcome: 26.8% in AVR vs 45.8% in CS (HR, 0.50; 95% CI, 0.40-0.63; *P* < .001)

AS, aortic stenosis; AVA, aortic valve area; AVAi, aortic valve area index; AV MG, aortic valve mean gradient; AV PV, aortic valve peak velocity; AVR, aortic valve replacement; CS, clinical surveillance; HR, hazard ratio; N/A, not applicable; SAVR, surgical aortic valve replacement; TAVR, transcatheter aortic valve replacement.

While these RCTs have shed light on the potential role of early AVR, it is important to note that management strategies used in rigorously conducted trials often do not represent real-world practice. In this context, Généreux et al^11^ performed a study-level meta-analysis of 4 RCTs and 12 observational studies including 5346 patients (2406 in the early AVR group and 2940 in the CS group). The weighted mean follow-up was ∼50 months in RCTs and 57 months in observational studies. Compared with CS, early intervention (predominantly SAVR) was associated with a lower incidence of all-cause mortality and cardiovascular mortality, a finding driven by observational studies.^11^ Additionally, early intervention was associated with a lower incidence of unplanned cardiovascular and heart failure-related hospitalizations. There was no difference in incidence of stroke between the 2 groups.^11^

Acknowledging the limitations inherent to study-level meta-analyses, the analysis by Généreux et al^11^ represents the most comprehensive comparing both strategies to date; however, some issues deserve further consideration. First, there was a considerable degree of heterogeneity between the studies in terms of intervention, extent of conversion to AVR in the CS group, and follow-up. Indeed, TAVR was the AVR modality in only 2 trials. There were also variations in the frequency of CS between the trials, which might have accounted for the differences in the crossover rates to AVR. For example, 47% and 28% of patients in the CS in the EARLY TAVR and EVOLVED trials, respectively, underwent conversion to AVR in 12 months. These rates are higher than the natural progression of asymptomatic severe AS, which suggests about 20% conversion to AVR due to symptoms per year.^12^ Second, the reduction in all-cause and cardiovascular mortality was driven by observational studies, which are prone to ascertainment and selection biases. Indeed, a prior study-level meta-analysis of the 4 RCTs showed a lack of reduction in all-cause and cardiovascular mortality with early intervention.^13^ Third, the definition of unplanned cardiovascular hospitalizations was different between the studies and included conversion to AVR in the EARLY TAVR trial.^10^ Fourth, the open-label design of these studies may have introduced bias, as patients in the CS would become hypervigilant to any symptoms once the diagnosis of severe AS was confirmed. In the EARLY TAVR trial, 98% of patients in the CS who converted to TAVR in the first 6 months were symptomatic (either New York Heart Association class III/IV symptoms or heart failure hospitalizations).^10^ Finally, the lack of patient-level data precluded further analyses to identify the subgroup of patients who benefit the most from early intervention. Notwithstanding these issues, the authors should be applauded for their work, which contributes to the existing body of literature regarding the prospects of early intervention among asymptomatic patients with severe AS.

For clinicians managing patients with asymptomatic severe AS, the question becomes “Should all patients undergo AVR once the echocardiographic criteria for severe AS are met, regardless of their symptom status?” With the current body of evidence, the answers to this may not be that simple. The benefits of AVR in asymptomatic severe AS should be balanced against operative risks associated with AVR and potential risk of prosthetic valve dysfunction. The latter may be relevant in younger patients given the lack of long-term outcome data beyond 10 years with TAVR. Of note, mortality among the early intervention group in EARLY TAVR was higher than the 3-year follow-up of the Evolut Low Risk Trial (8.4% vs 6.3%).^10^^,^^14^ Additional consideration should include patients with challenging anatomical characteristics (eg, small annulus) who were excluded from these trials. A heart team approach with patients’ preferences at the core should be considered in these scenarios. Ongoing RCTs (DANAVR [NCT03972644], EASY-AS [NCT04204915], and ESTIMATE [NCT02627391]) should help to better clarify the role of early intervention. Until then, it may not be unreasonable to recommend AVR for asymptomatic severe AS patients after shared decision making if patients are concerned about symptom development or future unplanned hospitalization.
